# A new species of the genus *Afissa* Dieke, 1947 (Coleoptera, Coccinellidae) from China

**DOI:** 10.3897/BDJ.13.e156613

**Published:** 2025-05-30

**Authors:** Lezhi Wang, Qifan Yang, Quan Zhang, Xingmin Wang

**Affiliations:** 1 Beijing No. 8 High School, Beijing, China Beijing No. 8 High School Beijing China; 2 College of Plant Protection, South China Agricultural University, Guangdong, China College of Plant Protection, South China Agricultural University Guangdong China

**Keywords:** Ladybird, Epilachnini, new species, China, taxonomy

## Abstract

**Background:**

Recent morphological and molecular studies have clarified some aspects of the taxonomy of *Afissa* Dieke, 1947, but many species remain unexamined or misclassified. Additional taxonomic work is essential to resolve the classification and distribution of Afissa species in Asia, particularly in China.

**New information:**

*Afissaxuexii* **sp. nov.** is described from Yunan and Guizhou Province, China. This new species is characteried by its typical bifurcated penis guide. Diagnosis, description and illustrations are provided.

## Introduction

The genus *Afissa* Dieke, 1947 (Coleoptera, Coccinellidae, Epilachnini) was established by Dieke (1947) to include the Eurasian species of *Epilachna* Chevrolat, 1837, which are characteriszed by the undivided sixth female segment and toothless claws, with *Coccinellaflavicollis* Thunberg, 1781 [=*Afissaflavicollis* (Thunberg, 1781)] designated as the type species. Li and Cook (1961), after examining the type species of *Epilachna* (*E.borealis* (Fabricius, 1775)) and *Afissa* (*A.flavicollis* (Thunberg, 1781)), noted that both type species shared the aforementioned characters and exhibit similar typical male genital structures; accordingly, Li and Cook (1961) reduced *Afissa* to synonymy with *Epilachna*. Szawaryn et al. (2015), based on molecular phylogenetic evidence, restored some *Asian* species of *Epilachna* to *Afissa* and synonymised the genus *Afissula* Kapur, 1985 with *Afissa*. Tomaszewska and Szawaryn (2016) further revised the tribe Epilachnini Mulsant, 1846, based on morphological characters and transferred several species from *Afissa* (*A.chapini* Dieke, 1947; *A.complicata* Dieke, 1947; *A.convexa* Dieke, 1947; *A.magna* Dieke, 1947; *A.militaris* Dieke, 1947; *A.quadricollis* Dieke, 1947; *A.subacuta* Dieke, 1947; *A.szechuana* Dieke, 1947) to the genus *Uniparodentata* Wang & Cao, 1993. In addition, several species from *Epilachna* (*E.parvula* Crotch, 1874; *E.sanscrita* Crotch, 1874; *E.plicata* Weise, 1889; *E.flavimarginalis* Hoàng, 1978; *E.ampliata* Pang & Mao, 1979; *E.max* Pang & Ślipiński, 2012) were transferred to *Afissa* and the diagnostic characters of *Afissa* were re-described.

Tomaszewska and Szawaryn (2016) proposed that most Asian species, formerly classified within *Epilachna*, likely belong to *Afissa*. In recent years, some *Epilachna* species have been re-assigned to *Afissa*, further supporting this view (Szawaryn 2018, Das et al. 2020, Das et al. 2023, Iqbal et al. 2024). The taxonomy of *Afissa* remains unresolved and further studies on this genus will help to clarify the confusion between *Afissa* and *Epilachna*.

In this study, we report a new species of *Afissa* from Yunnan and Guizhou Provinces, China and provide a description and illustration herein.

## Materials and methods

The specimens examined in this study were collected from Yunnan and Guizhou Provinces, China. All examined specimens are deposited in the Department of Entomology, South China Agricultural University (SCAU), Guangzhou, China.

The classification system follows Szawaryn et al. (2015). Adult morphological terminology used in this paper follows Ślipiński and Tomaszewska (2010). External structures were examined under a dissecting stereoscope (SteREO Discovery V20, Zeiss). The following measurements were recorded using a micrometer:

- **TL** total length, from apical margin of clypeus to apex of elytra;
- **TW** total width, across both elytra at widest part;
- **TH** total height, through the highest point of elytra to metaventrite;
- **HW** head width, including eyes;
- **PL** pronotal length, from the middle of the anterior margin to the base of the pronotum;
- **PW** pronotal width at widest part;
- **EL** elytral length, along the suture, from the apex to the base including the scutellum;
- **EW** elytral width, synonymous with TW.

Photographs of the habitus of specimens were taken with a digital camera (EOS 5D Mark IV, Canon), mounted on a focus stacking rail (WeMacro Rail), with Helicon Remote v. 3.9.12 utilised for image capture. The dissected male genitalia were cleaned in a 10% sodium hydroxide (NaOH) solution and transferred to neutral balsam for preservation. Genitalia were imaged using a digital camera (Axiocam 506 colour), connected to a microscope (Zeiss Imager M2), with ZEN 2.3 utilised for image capture. Helicon Focus v. 8.1.1 was used for image stacking. All images were further processed in Adobe Photoshop 2019.

## Taxon treatments

### Afissa

Dieke, 1947

852EEA83-42B3-58D6-A5D3-3AD484959740

Afissa Dieke, 1947: 113. Type species (original designation): *Coccinellaflavicollis* Thunberg, 1781. Type locality: East Indies. Synonymised with *Epilachna* Chevrolat in Dejean, 1837, by Li and Cook (1961). Resurrected from synonymy by Szawaryn et al. (2015).
Afissula Kapur, 1958. Type species (original designation): *Afissularana* (Kapur 1958). Mentioned in Jadwiszczak and Węgrzynowicz (2003); Kovár 2007; Ren et al. (2009). Synonymised by Szawaryn et al. (2015).
Epilachna Chevrolat in Dejean, 1837 (in part). Synonymised by Tomaszewska and Szawaryn (2016).

#### Diagnosis

*Afissa* can be reliably distinguished from all other Asian representatives and the remaining genera of Epilachnini by a unique combination of morphological characters: antenna longer than head width; coxites much longer than wide; mandibular incisor edge without teeth; lateral margins of elytra most often not or hardly visible from above (sometimes visible from above, but narrow); metanepisternum with simple, smooth inner margin; mid- and hind coxae with smooth hind margin.

### Afissa xuexii

Wang & Wang sp. nov.

F98C4932-027E-51D6-9E8E-76016E5F58CD

0F7F9FCD-CF91-4D18-B839-422222D7F9DF

#### Materials

- **Type status:** Holotype. **Occurrence:** recordedBy: Wang Xing-Min; individualCount: 1; sex: male; lifeStage: adult; occurrenceID: BE19C187-EF54-5DE9-85FF-ECA3C9DE85A7; **Taxon:** scientificName: Afissaxuexii; **Location:** country: China; stateProvince: Yunnan; county: Pingbian County; locality: Mount Dawei; verbatimElevation: 2100 m; **Event:** samplingProtocol: Sweep netting; eventDate: 11-19/10/2006; **Record Level:** collectionCode: Insects
- **Type status:** Paratype. **Occurrence:** recordedBy: Wang Xing-Min; individualCount: 16; sex: male; lifeStage: adult; occurrenceID: 5C9D6F4A-E372-5F39-B149-40C59A6964C9; **Taxon:** scientificName: Afissaxuexii; **Location:** country: China; stateProvince: Yunnan; county: Pingbian County; locality: Mount Dawei; verbatimElevation: 2100 m; **Event:** samplingProtocol: Sweep netting; eventDate: 11-19/10/2006; **Record Level:** collectionCode: Insects
- **Type status:** Paratype. **Occurrence:** recordedBy: Wang Xing-Min; individualCount: 10; sex: female; lifeStage: adult; occurrenceID: D77F8963-02CC-5456-B00F-5CC443AF6780; **Taxon:** scientificName: Afissaxuexii; **Location:** country: China; stateProvince: Yunnan; county: Pingbian County; locality: Mount Dawei; verbatimElevation: 2100 m; **Event:** samplingProtocol: Sweep netting; eventDate: 11-19/10/2006; **Record Level:** collectionCode: Insects
- **Type status:** Paratype. **Occurrence:** recordedBy: Ren S.X., Wang X.M., Chen X.S. & Hao J.Y.; individualCount: 11; sex: male; lifeStage: adult; occurrenceID: FE0D6A3E-53DB-52B1-A17E-06AA8970F09E; **Taxon:** scientificName: Afissaxuexii; **Location:** country: China; stateProvince: Yunnan; county: Pingbian County; locality: Mount Dajian; verbatimElevation: 2100 m; **Event:** samplingProtocol: Sweep netting; eventDate: 20-21/04/2008; **Record Level:** collectionCode: Insects
- **Type status:** Paratype. **Occurrence:** recordedBy: Ren S.X., Wang X.M., Chen X.S. & Hao J.Y.; individualCount: 12; sex: female; lifeStage: adult; occurrenceID: 4280CF0F-B3B1-5101-A688-6FA9BC4770BB; **Taxon:** scientificName: Afissaxuexii; **Location:** country: China; stateProvince: Yunnan; county: Pingbian County; locality: Mount Dajian; verbatimElevation: 2100 m; **Event:** samplingProtocol: Sweep netting; eventDate: 20-21/04/2008; **Record Level:** collectionCode: Insects
- **Type status:** Paratype. **Occurrence:** recordedBy: Wang X.M., Liang H.B., Hao J.Y. & Peng W.L.; individualCount: 8; sex: male; lifeStage: adult; occurrenceID: CE5122B9-D425-528C-8B5B-D488EE9FBF35; **Taxon:** scientificName: Afissaxuexii; **Location:** country: China; stateProvince: Yunnan; county: Cangyuan County; locality: Banhong Village, Nangun River; verbatimElevation: 1790 m; **Event:** samplingProtocol: Sweep netting; eventDate: 14-15/05/2008; **Record Level:** collectionCode: Insects
- **Type status:** Paratype. **Occurrence:** recordedBy: Wang X.M., Liang H.B., Hao J.Y. & Peng W.L.; individualCount: 4; sex: female; lifeStage: adult; occurrenceID: C5DA2DC2-44B0-5F3C-B6DE-CF123DC7BA77; **Taxon:** scientificName: Afissaxuexii; **Location:** country: China; stateProvince: Yunnan; county: Cangyuan County; locality: Banhong Village, Nangun River; verbatimElevation: 1790 m; **Event:** samplingProtocol: Sweep netting; eventDate: 14-15/05/2008; **Record Level:** collectionCode: Insects
- **Type status:** Paratype. **Occurrence:** recordedBy: Wang Xing-Min; individualCount: 3; sex: female; lifeStage: adult; occurrenceID: 4E1A4A11-67B8-5AD3-8816-804D84516C47; **Taxon:** scientificName: Afissaxuexii; **Location:** country: China; stateProvince: Guizhou; county: Zunyi City; locality: Xishui National Nature Reserve; verbatimElevation: 1016 m; verbatimCoordinates: 28°30′52″N, 106°28′18″E; decimalLatitude: 28.5144; decimalLongitude: 106.4717; **Event:** samplingProtocol: Sweep netting; eventDate: 18-22/07/2022; **Record Level:** collectionCode: Insects

#### Description

TL: 4.7-4.9 mm, TW: 3.5-3.8 mm, TH: 2-2.2 mm, TL/TW: 1.29-1.34; PL/PW: 2.18-2.2; EL/EW: 1.11-1.14.

**Figure 1.**
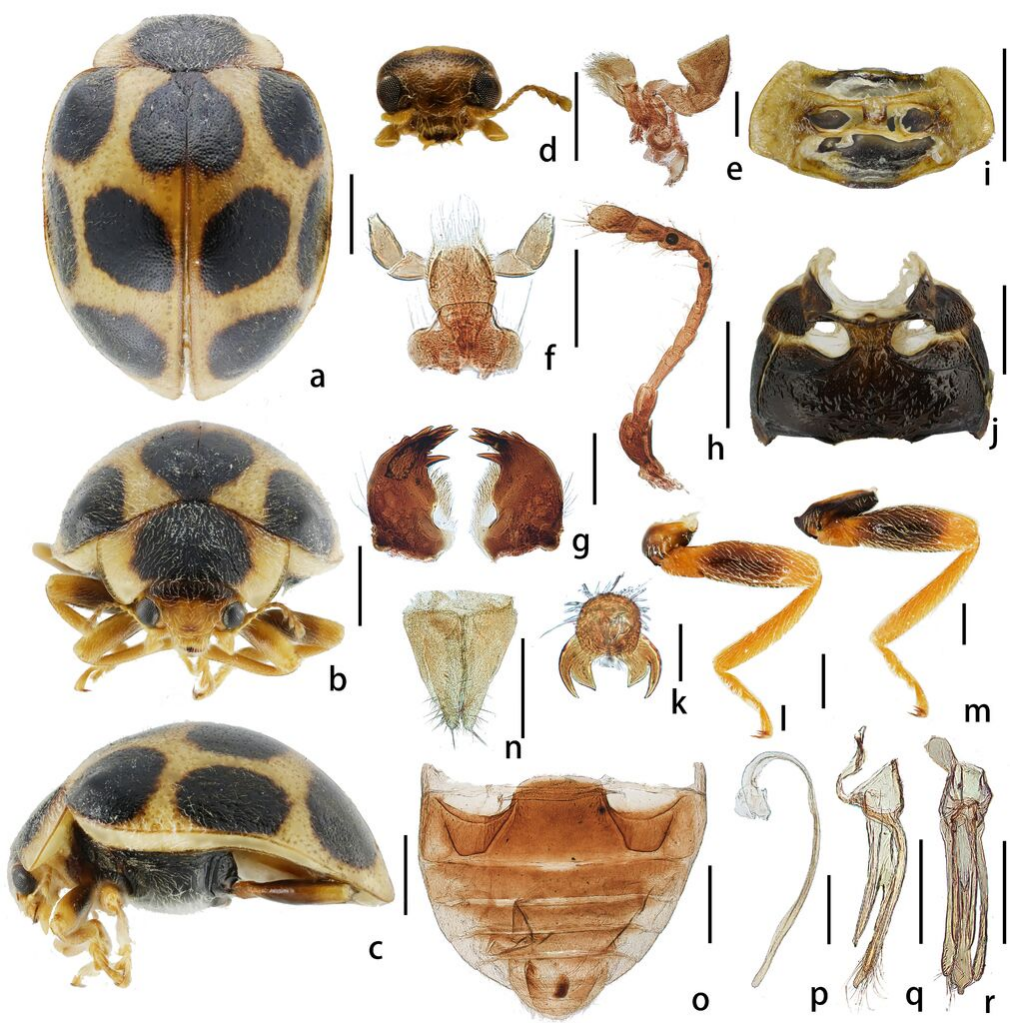
*Afissaxuexii* sp. nov. **a** dorsal view; **b** frontal view; **c** lateral view; **d** head frontal view; **e** maxilla; **f** labium; **g** left and right mandible; **h** antenna; **i** prothorax; **j** mesoventrite and metaventrite; **k** tarsal claws; **l** mid-leg; **m** hind leg; **n** female genitalia; **o** abdomen; **p** penis; **q** tegmen, lateral view; **r** tegmen, ventral view. Scale bars: a–c, d, h-j, l, m, o-r: 1.0 mm; e-g, k, n: 0.2 mm.

Body elongate-oval, moderately convex, with only a narrow outer margin flattened (Figs. 1a-c). Dorsum densely covered with white pubescence. Colour: Head yellow. Pronotum yellow with a large central black spot. Scutellum black. Elytra yellow with black spots. Prosternum yellow, meso- and metasternum black and epipleura yellow. Legs yellow; trochanters and 2/3 of femora blackish-brown.

Head relatively small, approximately 0.34 times the width of the body (Fig. 1d). Frons flat, with fine and dense punctures, inter-puncture distance 0.5–1.5 times their diameter; surface smooth and glossy, bearing short golden setae. Eyes relatively small, prominently projecting, with a rough surface and distinct small facets; interocular distance approximately 0.23 times the head width.

Pronotum transverse, arcuate lateral margins, slightly reflexed (Fig. 1b). Anterior angles not prominent, rounded and blunt; anterior margin arcuate and concave medially. Posterior angles rounded; posterior margin tightly fused to elytral base, width approximately 0.29 times body width (body width/pronotal width = 3.45). Surface with punctures similar in size to those on the head, smooth and glossy, inter-puncture distance 0.5–1.0 times their diameter.

Elytra strongly vaulted, smooth and glossy, densely punctured (Fig. 1a). Punctures small, indistinct and varying in size. Each elytron with five black spots arranged in 2-2-1 pattern. Black spot near scutellum fused at suture.

Prosternum T-shaped, with a quadrate process, lacking prosternal process, surface rough and densely covered with fine setae (Fig. 1i). Mesosternum trapezoidal, with a slight longitudinal elevation at the centre, anterior margin slightly concave medially, surface rough and densely covered with fine setae (Fig. 1j). Metasternum wide, with a distinct median discrimen, longitudinally concave near the median discrimen, with central elevation on both sides; surface finely punctate and densely covered with short fine setae, inter-puncture distance 1.0–2.5 times their diameter. Abdomen with nearly complete abdominal postcoxal lines, posterior margin almost reaching the hind margin of the first abdominal segment (Fig. 1o).

Tarsal claws with two terminal teeth, inner tooth non-opposing, lacking basal tooth (Fig. 1k).

Male genitalia. Penis slender, curved in circular shape, with a short, expanded penis capsule (Fig. 1p). Distal 1/3 of the penis slightly enlarged and rounded at apex. In lateral view (Fig. 1q), distal half of the penis guide splits into two parts, part near the parameres longer. Parameres slender, arcuate, slightly shorter than the penis guide, with sparse setae at apex. In ventral view (Fig. 1r), shorter part of the penis guide noticeably wider than longer part, the latter with a finger-like process at apex.

Female genitalia. Coxites subtriangular, densely setose apically. Inner margins smooth, without depressions. Styli distinct, each bearing an apical seta (Fig. 1n).

#### Diagnosis

*Afissaxuexii* closely resembles *A.expansa* (Dieke, 1947), *A.hydrangeae* Pang & Mao, 1979, *A.kambaitana* (Bielawski, 1966) and *A.anhweiana* (Dieke, 1947) in elytral colouration and pattern and their distributions are geographically proximate. However, *A.xuexii* can be easily distinguished from these species by its bifurcate penis guide and the elongate-oval body shape. Bifurcate penis guide is a rare and distinctive diagnostic character within *Afissa*.

#### Etymology

The new species is named after Xuexi, an institution that provided valuable support and inspiration in the first author’s study of insect taxonomy.

#### Distribution

China (Yunnan, Guizhou).

## Discussion

Pang et al. (2012) provided the comprehensive taxonomic study on the genus *Epilachna* in China, recording over 100 species from China. However, following the revision of Epilachnini by Szawaryn et al. (2015) and Tomaszewska and Szawaryn (2016), only very few species were correctly transferred to *Afissa*, while the majority remain unexamined. Tomaszewska and Szawaryn (2016) suggested that a considerable number of Asian species previously placed in *Epilachna* may actually belong to *Afissa*.

This study represents the only recent contribution describing new species of *Afissa* and the discovery further supports the diagnostic characters shared by *Afissa*. In recent years, several taxonomic revisions have been conducted: Das et al. (2020) revised *A.gibbera* (Crotch, 1874), *A.mystica* (Mulsant, 1850) and *A.undecimspilota* (Hope, 1831); Das et al. (2023) revised *A.langpingensis* (Zeng & Yang, 1996) and *A.sureilica* Kapur, 1961; and Iqbal et al. (2024) revised *A.cuonaensis* (Pang & Mao, 1977), *A.schawalleri* (Canepari, 1997) and *A.similbengalica* (Canepari, 2012). Although some of these species are known from China, none of the recent studies re-examined any Chinese specimens. Therefore, a thorough revision of *Epilachna* species in China is a crucial task in the morphological taxonomy of Chinese Coccinellidae.

## Supplementary Material

XML Treatment for Afissa

XML Treatment for Afissa xuexii
